# May we learn a useful lesson from prevention rules against severe acute respiratory coronavirus virus 2 (SARS-CoV-2)?

**DOI:** 10.1017/ice.2020.1228

**Published:** 2020-09-23

**Authors:** Silvia Corbellini, Maria Antonia De Francesco, Arnaldo Caruso

**Affiliations:** Department of Molecular and Translational Medicine, Institute of Microbiology, University of Brescia-ASST Spedali Civili di Brescia, Brescia, Italy

*To the Editor—*The emergence and diffusion of oxacillin-resistant *Staphylococcus aureus* (MRSA) constitutes an important problem for public health. Data from European countries reported a trend with an increasing MRSA prevalence from the north to the south of the continent: <5% of MRSA has been isolated from invasive infections in north of Europe compared with 25%–50% in the south of Europe.^1^

This gram-positive bacterium is generally found as part of commensal flora in the nasal mucosa in 20%–40% of the population and just these people, who are asymptomatic carriers, have an increased risk to acquire a subsequent infection in addition to representing an important source of person-to-person transmission. In particular, hospital and healthcare settings represent a favorable environment that predispose to infection because of a high antibiotic selection pressure, the use of invasive procedures, and the presence of critically ill patients. For these reasons, MRSA is now endemic in many hospitals worldwide, and infection control measures are needed to prevent its transmission, especially considering the risk of development of glycopeptide-resistant *S. aureus* strains.

Hospital control of endemic MRSA has been based on standard precautions such as isolation/cohorting, hand hygiene, patient decolonization, and appropriate use of antibiotic (antibiotic stewardship). Intensive care units of Spedali Civili’s Hospital of Brescia has implemented active surveillance cultures to identify patients who acquire MRSA during hospitalization. This surveillance involves nasal swabs for the screening of patients at the time of hospital admission to identify asymptomatic carriers, followed by periodic screening every 3 days.

One of these intensive care units became a coronavirus disease 2019 (COVID-19) ward during the pandemic, and we analyzed whether the higher compliance to the use of personal protective equipment (PPE, eg, gloves, coveralls, face mask and boots) by all the hospital staff had an impact on the prevalence of MRSA acquisition during patients hospitalization.

It is well known that healthcare workers can transmit infections such as tuberculosis, varicella and influenza by the airborne route,^2^ but it less well known that airborne and other ways of transmission may occur with some bacterial pathogens. In particular, the use of face masks prevents pathogen transmission from the wearer to other people and reduces hand-to-face contact and facial contact with droplets.^3^

In our analysis, we compared the MRSA detection after 48 hours following hospital admission during January–August 2020 versus January–August 2019. As shown in Table 1, we observed a statistically significant reduction in the prevalence of nosocomially acquired MRSA (2% vs 14%; *P* < .0001). This decrease was always statistically significant for all the months analyzed except January, when the implementation of PPE in the absence of COVID-19 was not present (Table 1). This finding is not surprising, since a previous study showed that a healthcare worker, who did not wear a mask and who was a nasal carrier of MRSA, induced a 40-fold increase in MRSA dispersion.^4^

**Table 1.** Trend of MRSA Detection During the Study Period

	2019	2020	
Year	No. of Positive/Total (%)	No. of Positive/Total (%)	*P* Value
January	9/103 (8)	4/134 (3)	.08
February	10/102 (9)	2/126 (2)	.01
March	18/135 (12)	5/274 (2)	.0001
April	13/108 (11)	7/269 (3)	.01
May	25/97 (20)	4/193 (2)	.0001
June	21/83 (20)	2/178 (1)	.0001
July	17/97 (15)	0/183 (0)	.0001
August	21/107 (16)	8/153 (5)	.01
Total	134/832 (14)	32/1510 (2)	.0001

Although our preliminary data need to be confirmed by larger studies, our observation suggests implementation of PPE as a strong preventive strategy to control hospital*-*acquired MRSA infection.
